# A Role for Adenosine Deaminase in *Drosophila* Larval Development

**DOI:** 10.1371/journal.pbio.0030201

**Published:** 2005-05-24

**Authors:** Tomas Dolezal, Eva Dolezelova, Michal Zurovec, Peter J Bryant

**Affiliations:** **1**Developmental Biology Center, University of California, Irvine, California, United States of America; **2**Institute of Entomology and University of South Bohemia, Ceske Budejovice, Czech Republic; Stanford University School of MedicineUnited States of America

## Abstract

Adenosine deaminase (ADA) is an enzyme present in all organisms that catalyzes the irreversible deamination of adenosine and deoxyadenosine to inosine and deoxyinosine. Both adenosine and deoxyadenosine are biologically active purines that can have a deep impact on cellular physiology; notably, ADA deficiency in humans causes severe combined immunodeficiency. We have established a *Drosophila* model to study the effects of altered adenosine levels in vivo by genetic elimination of adenosine deaminase-related growth factor-A (ADGF-A), which has ADA activity and is expressed in the gut and hematopoietic organ. Here we show that the hemocytes (blood cells) are the main regulator of adenosine in the *Drosophila* larva, as was speculated previously for mammals. The elevated level of adenosine in the hemolymph due to lack of ADGF-A leads to apparently inconsistent phenotypic effects: precocious metamorphic changes including differentiation of macrophage-like cells and fat body disintegration on one hand, and delay of development with block of pupariation on the other. The block of pupariation appears to involve signaling through the adenosine receptor (AdoR), but fat body disintegration, which is promoted by action of the hemocytes, seems to be independent of the AdoR. The existence of such an independent mechanism has also been suggested in mammals.

## Abstract 2

Adenosine deaminase is critically important to survival; congenital deficiency in humans leads to severe immunodeficiency. Here, the authors demonstrate that adenosine deaminase deficiency in flies results in severe developmental defects.

## Introduction

Adenosine deaminase (ADA) is an enzyme present in all organisms that catalyzes the irreversible deamination of adenosine and deoxyadenosine to inosine and deoxyinosine. It is a critically important enzyme for human survival because its congenital absence causes severe combined immunodeficiency disease (SCID). ADA deficiency accounts for about 20% of all types of SCID [1]. It is one of the most severe human immunodeficiencies and is associated with depletion of all three major categories of lymphocytes: T cells, B cells, and natural killer cells, resulting in impaired cellular immunity and decreased production of immunoglobulins [2]. Without intervention, the affected individuals die from opportunistic infections within the first few months of life.

ADA occurs as a soluble monomer in all human cells, but also exists as “ecto-ADA,” bound to the membrane glycoprotein CD26/dipeptidyl peptidase IV, and it has been suggested that this form of ADA regulates extracellular adenosine levels [3]. ADA deficiency is accompanied by greatly elevated levels of the ADA substrates adenosine and deoxyadenosine, both of which are biologically active purines that can have a deep impact on cellular physiology. Adenosine is not just a metabolite; it is also a signaling molecule that regulates numerous cellular functions by binding to G protein-coupled adenosine receptors (A1, A2a, A2b, and A3 in mammals) that can regulate intracellular cyclic adenosine monophosphate [4]. Deoxyadenosine is a cytotoxic metabolite released by various cell populations that undergo programmed cell death; it can kill cells through a mechanism that includes disturbances in deoxynucleotide metabolism [5].

Extracellular adenosine is now considered an important stress hormone that is released in excessive amounts in the vicinity of immune cells during both systemic and cellular stress [6]. The predominant source of extracellular adenosine during systemic activation of the stress system is the sympathetic nervous system [7]. Specific inflammatory stimuli such as bacterial products are also capable of triggering adenosine release from immune cells [8]. These data are in line with evidence demonstrating a dramatic increase in extracellular adenosine levels under conditions associated with multiple organ failure, which is the cause of 50%–80% of all deaths in surgical intensive care units [6].

ADA is not the only adenosine deaminase in mammalian cells. Recently, the *cat eye syndrome critical region protein 1 (CECR1)* gene was identified and shown to encode a protein representing a subfamily of proteins related to but distinct from classical ADAs [9]. The duplication of a small region of chromosome 22 containing this gene is associated with “cat eye syndrome,” a disorder characterized by hypoplastic kidneys, congenital heart malformation, and anomalous pulmonary venous connections. The founding member of this subfamily is encoded by *insect-derived growth factor* *(IDGF)* [10], and homologs have been described in various organisms [11–14].

We have previously found six *Drosophila* genes with sequence similarity to the *CECR1* subfamily [15]. Their products are mitogenic on *Drosophila* cells, and at least two of them (ADGF-A and ADGF-D) exhibit strong ADA activity, which is necessary for their mitogenic function. We therefore named them adenosine deaminase-related growth factors (ADGFs). We also demonstrated that adenosine functions as a negative signal for cell proliferation and concluded that ADGFs stimulate cell growth in vitro by depletion of extracellular adenosine [16]. *Drosophila* also contains a gene, termed *Ada,* with sequence similarity to human *ADA,* but as we have previously shown the product of this gene is most likely not an active ADA [16].

In this report we show that a null mutation in *Drosophila ADGF-A* gene leads to dramatically increased levels of adenosine and deoxyadenosine in the larval hemolymph. This increase leads to larval death associated with the disintegration of fat body and the development of melanotic tumors. We present a detailed analysis of the hematopoietic defects associated with the *adgf-a* mutation, show a genetic interaction of this mutation with signaling through the *Drosophila* adenosine receptor (AdoR, encoded by the gene *CG9753*) and with regulation of premetamorphic changes by ecdysone, as well as a genetic interaction of ADGF-A with a major innate immunity regulator—the Toll signaling pathway.

## Results

### Mutation in the *ADGF-A* Gene Causes Larval Death and Melanotic Tumors

We produced mutations in five of the six *ADGF* genes by homologous recombination mutagenesis [17] and showed that loss of the most abundantly expressed gene, *ADGF-A,* leads to death in the larval or pupal stage. Under optimal conditions (20–30 isolated homozygous larvae per vial), about 60% of larvae homozygous for the *adgf-a* mutation reach the third instar. Development during the third larval instar is significantly delayed, and wandering homozygous larvae usually appear 2 d after their heterozygous siblings, which start wandering at about 5 d of development. Some homozygous third-instar larvae can be found alive in the vial even after 10 d of development. Mutant third-instar larvae show fat body disintegration (Figure 1A and 1B) and multiple melanotic tumors (Figure 1C), predominantly in the caudal part of the body and accompanied by disintegration of the fat body. Melanization of the lymph glands was never observed in these larvae, and the imaginal discs and brain appear normal. Less than 30% of homozygotes eventually pupate. Homozygous pupae usually die soon after pupariation; in some cases they develop normal head and thorax imaginal structures; however, abdominal parts usually do not develop. There is also an abnormal curvature (to the right) of the pupal abdomen (Figure 1D). Less than 2% of mutant pupae develop normally and eventually emerge as adults without any obvious abnormalities besides the abdominal curvature; some of them are sterile.

**Figure 1:**
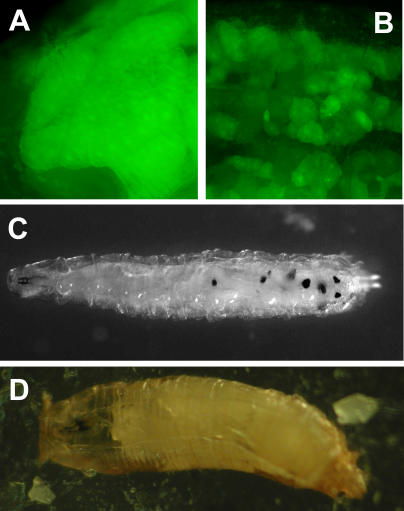
adgf-a Mutant Phenotype (A and B) Fat body disintegration visualized by GFP expression driven by *Cg-Gal4* driver in the fat body. While *adgf-a/+* heterozygous third instar larvae have normal flat layers of fat body (A),*adgf-a* mutant showed extensive fat body disintegration into small pieces of tissue (B). (C) Multiple melanotic tumors present in *adgf-a* mutant third-instar larva. (D) An *adgf-a* mutant pupa with typical abdominal curvature.

To confirm that the mutant phenotype is caused solely by a mutation in the *ADGF-A* gene, we created transgenic flies carrying the *ADGF-A* gene under a heat-shock promoter *(HS-ADGF-A)*. The *adgf-a* homozygous flies carrying the *HS-ADGF-A* construct showed survival rates significantly higher than *adgf-a* even without heat shock, probably due to leaky expression of the *HS-ADGF-A* construct (Figure 2A). However, while non-heat shocked animals still produced many melanotic tumors, only 22% of animals that were heat shocked as late embryos/early first instar developed these tumors (Figure 2B). This result confirms that the mutant phenotype is caused by the mutation in the *ADGF-A* gene. This conclusion is further supported by the even more efficient rescue achieved by expression of transgenically provided ADGF-A in the lymph glands using the Gal4/UAS system (see below).

**Figure 2:**
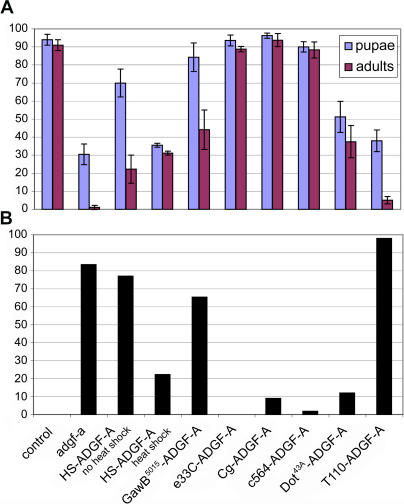
Rescue of the *adgf-a* Mutant Phenotype by Expression of ADGF-A in Different Tissues (A) Percentage of pupae (blue bars) and adult flies (purple bars) demonstrating the larval and pupal survival, respectively, of the *adgf-a* mutant flies rescued by expression of transgenic ADGF-A in different tissues. Along the *x*-axis (which is shared with [B]), the rescue experiments are shown (marked by the *Gal4* driver used for expression of ADGF-A except for first three sets of bars—the first set presents only an *adgf-a* mutant, the second an *adgf-a* mutant carrying *HS-ADGF-A* construct without heat shock, and the third with heat shock) and the *y*-axis represents percentage of pupae and adult flies out of the total number of transferred first-instar larvae of particular genotype. Each experiment was repeated at least four times (with 20–30 animals in each vial) and the standard error is shown. (B) Percentage of late third-instar larvae with melanotic tumors. The *x*-axis is shared with (A) (described above). The *y*-axis shows the percentage of larvae with tumors out of all larvae of each genotype examined for (A).

### The *adgf-a* Mutant Phenotype Is Associated with Elevated Levels of Adenosine and/or Deoxyadenosine

Using liquid chromatography and mass spectrometry of deproteinated hemolymph samples, we measured adenosine concentrations in hemolymph of mutant and wild-type third-instar larvae. The adenosine concentration in the *adgf-a* mutant was 1.14 ± 0.26 μM compared to less than 0.08 μM in the wild type, and the deoxyadenosine concentration in mutants was 1.66 ± 0.99 μM compared to an undetectable level in the wild type.

### The Catalytic Activity of ADGF-A Is Required for Its Function

To test whether the function of ADGF-A in vivo is also dependent on its catalytic activity, we produced two versions of the *UAS-ADGF-A* construct [18]: one carrying wild-type cDNA of ADGF-A and one carrying an ADGF-A cDNA with a mutation causing a substitution of two amino acids (H386G and A387E) in the catalytic domain [16]. Two different lines carrying the wild-type *UAS-ADGF-A* expression construct together with an *Actin-Gal4* driver (providing ubiquitous expression) both completely rescued the mutant phenotype, whereas larvae with *UAS-ADGF-A* but without the driver showed the typical mutant phenotype. However, neither of the two lines carrying the mutated version of the *UAS-ADGF-A* (producing full-length protein detected by anti-myc antibody; see Materials and Methods) showed any rescue of the mutant phenotype. This result therefore demonstrates that the catalytic activity of ADGF-A is required for its function in vivo.

### Hemocyte Development Is Affected in the *adgf-a* Mutant

We investigated the number and morphology of hemocytes (blood cells) in the hemolymph of the *adgf-a* late third-instar larvae (Figures 3 and 4). These larvae contain an average of seven-fold more hemocytes in circulation than wild-type larvae (Figure 3). In contrast to normal larval plasmatocytes, which remain rounded after settling down on the substrate (Figure 4A), most of the cells in the *adgf-a* mutant (more than 75%) are strongly adhesive and, after they are deposited in a drop of hemolymph on a microscope slide, develop filamentous and membranous extensions (Figure 4B–4D). An average of 7% of hemocytes in the *adgf-a* mutant are lamellocytes (Figures 3 and 4E), large flat cells that are not present in circulation of wild-type larvae under normal conditions [19]. Crystal cells were also detected in excess, with mutant larvae carrying several hundred while there are fewer than a hundred of these cells in the wild type (Figure 5). The lymph glands normally do not release hemocytes into the hemolymph before metamorphosis [20]; instead, they are released during metamorphosis when the lymph glands disperse [19]. However, the lymph glands of *adgf-a* mutant larvae are already dispersed in the late third instar. This process is similar to normal metamorphic changes, in which the hemocytes are first released from the front lobes, and the posterior lobes disperse later.

**Figure 3:**
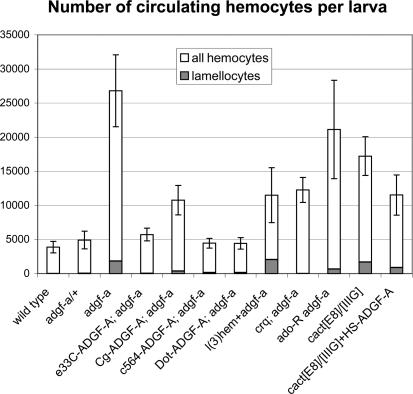
Number of Circulating Hemocytes in Late Third-Instar Larvae Genotypes are shown along the *x*-axis, and the number of hemocytes/larva along the *y*-axis. Each bar shows the number of all circulating hemocytes, and the gray part of the bars represent the lamellocyte population. Each count was repeated five to ten times and the standard error is shown.

**Figure 4:**
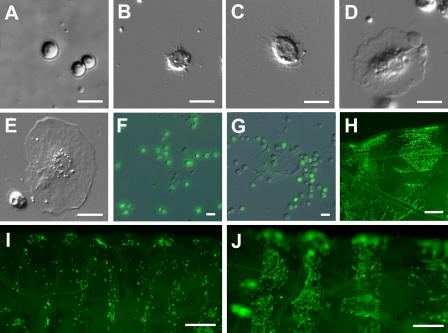
Hemocyte Abnormalities in *adgf-a* Mutant Larvae (A–E) Differential interference contrast microscopy of living circulating hemocytes (magnification 200×; scale bar, 10 μm). Round, nonadhesive plasmatocytes from wild-type larva (A). Hemocytes from the *adgf-a* mutant developing filamentous extensions (B and C) or membranous extension surrounding the cell (D). Large flat lamellocyte from the *adgf-a* mutant (E). (F and G) Differential interference contrast and fluorescent microscopy (merged image) of living circulating hemocytes stained by the *Hml-GFP* marker (magnification 100×; scale bar, 10 μm). While most of the cells from wild-type larvae are GFP-positive (F), just few of the cells from late third instar *adgf-a* larvae are stained by GFP at this stage (G). (H–J) Fluorescence microscopy of living larvae with Hml-GFP stained hemocytes (magnification 40×; scale bar, 100 μm). Posterior part of late third-instar wild-type larva (H). Middle sections of early third-instar larvae of wild type (I) and *adgf-a* mutant (J).

**Figure 5:**
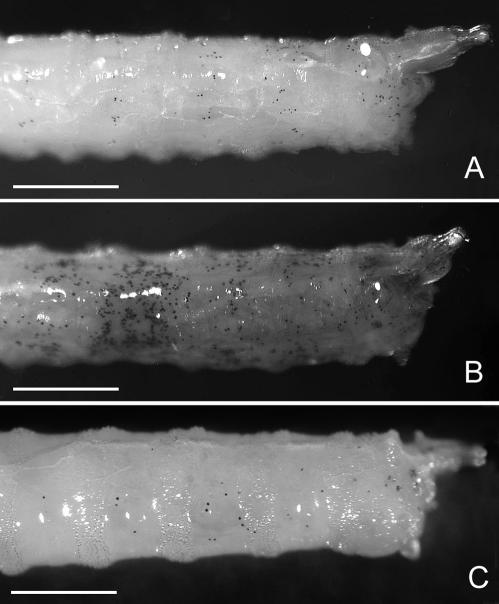
Crystal Cells in Late Third Instar Larvae Crystal cells were visualized by heating larvae of different genotypes at 60 °C for 10 min [46]. (A) Wild-type larva, (B) *adgf-a* single mutant, (C) *adoR adgf-a* double mutant (scale bar, 0.5 mm).

To analyze hemocytes in living larvae, we used the *Hemolectin* marker *(Hml)* [21]. We compared the number and distribution of hemocytes stained by GFP in flies carrying *hml-Gal4 UAS-GFP* in wild-type and mutant backgrounds. While there are relatively few hemocytes, mostly free-floating in the hemolymph, in early third-instar wild-type larvae (see Figure 4I), a much higher number of hemocytes, which are mostly attached to the tissues under the integument (described as sessile hemocytes in [19]), was observed in mutant larvae (see Figure 4J). A similar behavior was detected later in wild-type larvae, toward the end of the third instar (see Figure 4H). At this stage, the *Hml* marker disappeared from the most of the hemocytes in mutants (see Figure 4F and 4G).

### The *adgf-a* Mutant Phenotype Is Rescued by Expression of ADGF-A in the Lymph Glands

To distinguish which tissues require ADGF-A expression for proper development, we tested for rescue of *adgf-a* lethality by expressing ADGF-A in specific subsets of larval tissues. A transgenic line carrying the *UAS-ADGF-A* construct on Chromosome II was crossed to lines expressing the Gal4 driver [18] in different tissues (Table 1). Since ADGF-A is normally expressed in the larval lymph glands [16], and the mutant phenotype is characterized by abnormal hemocyte development, special consideration was given to lines expressing the Gal4 driver in the lymph glands and/or circulating hemocytes. No line expressing the Gal4 driver exclusively in the lymph glands has been reported, so we used a combination of lines sharing in common the feature of Gal4 driver expression in the lymph glands. The results (see Figure 2 and Table 1) clearly demonstrate that expression of ADGF-A in the lymph glands (driven by *Cg-Gal4,* *e33C-Gal4,* or *c564-Gal4*), but not in any other tissue examined, is necessary and sufficient to fully rescue the *adgf-a* lethality. In *e33C-Gal4/UAS-ADGF-A,* strong expression of ADGF-A in all lobes of developing lymph glands (but not in circulating hemocytes) reduces the number of hemocytes in the hemolymph to almost normal levels (see Figure 3). The number of hemocytes is also reduced, but to a lesser extent in larvae rescued by *Cg-Gal4/UAS-ADGF-A*. However, when assayed by survival rate and melanotic tumor formation, the rescue by *Cg-Gal4* is full and similar to that of *e33C-Gal4* (see Figure 2). The difference in effectiveness may be explained by the different expression patterns of the drivers. *Cg-Gal4* is expressed only in certain compartments of lymph gland lobes containing relatively mature hemocytes, and strongly in most circulating hemocytes [22, 23]. The *C564-Gal4* driver is not expressed as strongly as *e33C-Gal4,* but is still uniformly expressed in the lymph glands; it also fully rescued the mutant phenotype. We have tried two different insertions of the *Dot-Gal4* construct. The *Dot-Gal4^11C^* on Chromosome II, which shows weak expression [24], did not rescue the phenotype, but a *Dot-Gal4^43A^* insertion on Chromosome X, which shows stronger expression, rescued approximately half of the mutant animals (Figure 2). Nearly all rescued individuals were males, suggesting that expression of the Gal4 driver was influenced by X-chromosome dosage compensation, and expression in females heterozygous for *Dot-Gal4* was not strong enough for rescue.

**Table 1:**
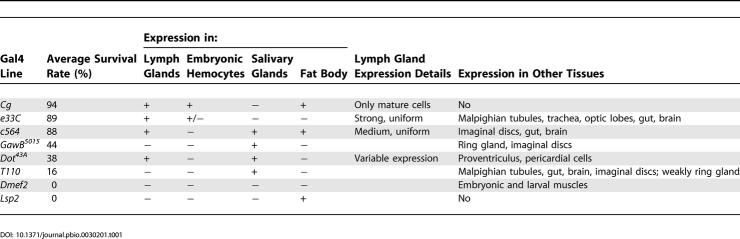
Gal4 Drivers—Expression Pattern and Rescue of the *adgf-a* Phenotype

Expression of ADGF-A in salivary glands and fat body (as well as in other tissues) is not required for full rescue, as demonstrated by use of the *Cg-Gal4,* *Dot-Gal4,* but especially by *e33C-Gal4* driver, and is also not sufficient to rescue the phenotype at all, as demonstrated by *T110-Gal4* and *Lsp2-Gal4* (Table 1).

Since ADGF-A is strongly expressed in embryonic mesoderm [16], we have tried to rescue the phenotype by the expression of ADGF-A in embryonic and larval muscle cells using the *Dmef2-Gal4* driver [25]. No rescue of the phenotype, including body shape of escaping pupae, was observed.

The only line showing significant (but not complete) rescue of *adgf-a* survival without expression in the lymph glands was *GawB^5015^* (see Figure 2), which expresses the Gal4 driver very strongly and specifically in the ring gland and salivary glands (as well as very weak and spotty expression in imaginal discs [unpublished data]). However, expression of ADGF-A driven by *GawB^5015^* does not prevent the formation of melanotic tumors (see Figure 2B).

### Ablation of Hemocytes in Mutant Larvae Reduces Fat Body Disintegration and Melanotic Tumor Formation

The *l(3)hematopoiesis missing* *(l[3]hem)* mutation reduces cell division in larval proliferating tissues and thus dramatically reduces the number of hemocytes in larvae. It also suppresses the hemocyte overproliferation and associated defects observed in the *hopscotch^Tumorous-lethal^* mutant [26]. We therefore used the *l(3)hem^1^* mutation to test whether the reduction of hemocyte number in the *adgf-a* mutant affects the phenotype. We recombined this mutation onto the chromosome containing the *adgf-a* mutation and found that in homozygous *l(3)hem^1^,* *adgf-a* double mutants the number of hemocytes is significantly reduced compared to the *adgf-a* single mutants (see Figure 3). Furthermore, while 90% of *adgf-a* mutant larvae showed disintegration of fat body, only 40% of *l(3)hem^1^,* *adgf-a* double mutants (total number of counted animals was 82) show the disintegration (Figure 6A). Similarly, melanotic tumor formation is significantly suppressed by *l(3)hem^1^*, with only 55% of double mutants showing melanotic tumors compared to more than 83% in *adgf-a* (Figure 6A). However, the delay in development and block of pupariation (Figure 6B), as well as the pupal body shape, were not influenced by this mutation. This shows that the effect on hemocyte development is related to only one other aspect of the *adgf-a* phenotype—namely, fat body disintegration—and the developmental arrest of *adgf-a* mutants is probably independent of this process.

**Figure 6:**
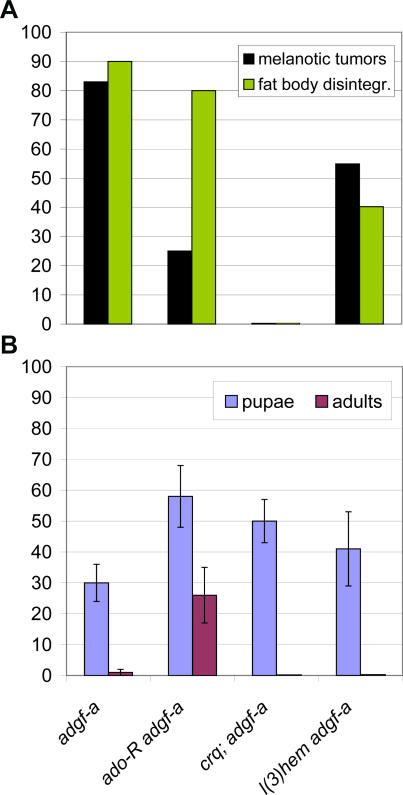
Suppression of the *adgf-a* Mutant Phenotype by Mutations in Other Genes (A) Percentage of late third-instar larvae with melanotic tumors (black bars) and fat body disintegration (green bars). The *x*-axis (which is shared with [B]), shows the genotype. The *y*-axis shows the percentage of larvae with tumors and fat body disintegration. (B) Survival rate of double mutants compared to single *adgf-a* mutant. The *y*-axis shows the percentage of the pupae (blue bars) and adult flies (purple bars) demonstrating the larval and pupal survival, respectively. Each experiment was repeated at least four times (with 20–30 animals in each vial) and the standard error is shown.

### Block in Activation of Macrophages Suppresses Disintegration of Fat Body

Previous results suggest that fat body disintegration might be caused by the action of hemocytes. Embryonic macrophages express the scavenger receptor encoded by *croquemort* *(crq),* which allows them to bind and remove apoptotic corpses [27]. We therefore tested whether a mutation in the *crq* gene would block the suggested interaction between hemocytes and fat body in *adgf-a* mutant larvae. We used the mutation *crq^KG01679^,* caused by a P-element insertion in the first untranslated exon of *crq,* which leads to pupal lethality. The number of crystal cells was not increased and lamellocytes were not detected in *crq, adgf-a* double mutants (see Figure 3). The double mutants showed a lower number of circulating hemocytes than the single mutant, but there was still a significant increase in this number compared to wild type (see Figure 3), and the cells showed increased clumping. None of the double-mutant larvae showed either disintegration of fat body or melanotic tumor formation (Figure 6A). Even the *adgf-a* mutant larvae heterozygous for the *crq* mutation *(crq/CyO GFP; adgf-a/adgf-a)* showed significant suppression of the fat body disintegration, with most of the tissue staying compact in bigger pieces and never disintegrating to single adipose cells; melanotic tumors were rarely observed. This shows that the block of the putative interaction between fat body and macrophage-like cells (which are still present in double mutants) suppresses the fat body disintegration, further strengthening the hypothesis that the disintegration is caused by hemocytes. In addition, the absence of lamellocytes and the normal number of crystal cells in the double mutant strongly suggest that the differentiation of these cells and thus melanotic tumor formation is a secondary reaction to fat body disintegration, rather than a primary effect of the *adgf-a* mutation.

### Mutation in a Putative Adenosine Receptor Suppresses the Block of Pupariation in *adgf-a*

We have identified a putative homolog of the mammalian adenosine receptor family in the *Drosophila* genome, AdoR, and produced a null mutation in this gene using homologous recombination (*adoR;* ED, unpublished data). The *adoR* mutants are fully viable. We used this mutant to test the hypothesis that the increased level of adenosine in the *adgf-a* mutant contributes to the mutant phenotype by its effect on signaling through the adenosine receptor. The results show that introducing the *adoR* mutation into the *adgf-a* background significantly increases pupariation, as well as adult emerging rate, compared to the *adgf-a* single mutant (Figure 6B). When the earlier lethality was avoided by picking up larvae after molt to the third instar, the pupariation rate of *adoR, adgf-a* double mutant was comparable to wild type as well as to the single *adgf-a* mutant treated with ecdysone (Figure 7A). Development during the third instar is much less delayed in the double mutant, with most of the larvae pupariating within 1 d after their heterozygous siblings (Figure 7A).

**Figure 7:**
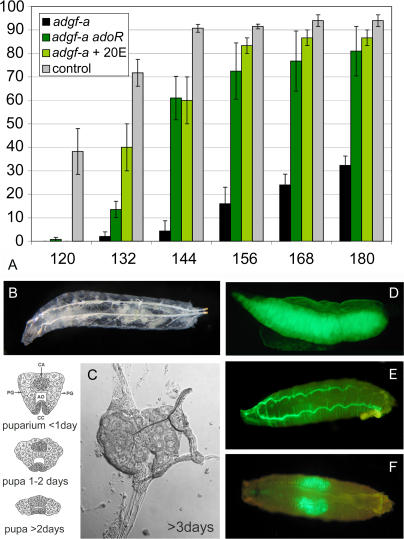
Ecdysone Regulation of Development in *adgf-a* (A) Larvae of different genotypes were collected after L2/L3 molt, and the number of puparia was counted at different time points (*x*-axis: hours after egg laying). The *y*-axis shows the percentage of puparia out of all collected third-instar larvae (three vials each with 30 animals; the standard error is shown). (B and C) Ring gland morphology in arrested *adgf-a* larvae. Approximately 8-d old mutant larva (i.e., 3 d after normal pupariation) with very extensive fat body disintegration (note the transparency of larva in the middle part with small white pieces of fat body) (B). The ring gland dissected from this larva (C) shows morphology of the normal ring gland before the degenerative changes of prothoracic gland starts (compare to schematic diagram to the left of [C], from [28]). (D–F) Expression of GFP-marked glue protein (SgsΔ3-GFP) in salivary gland of the *adgf-a* mutant larvae and pupae. All late third-instar larvae express the glue protein as shown on dissected salivary gland (D). Some mutants show typical expulsion from the glands with GFP totally external to the puparial case (E), while others do not expel glue proteins even after puparium formation (F).

The *adoR* mutation also significantly reduced melanotic tumor formation in the *adgf-a* mutant (see Figure 6A), but disintegration of the fat body appeared at the same rate as in the single mutant (see Figure 6A). While the number of macrophage-like cells in circulation is not significantly changed in the double mutant, the number of lamellocytes is decreased (see Figure 3), but the number of crystal cells is normal (see Figure 5A and 5C).

These results demonstrate that adenosine signaling through the adenosine receptor is involved in the developmental arrest of *adgf-a* mutant, but that it does not play a role in fat body disintegration and macrophage differentiation.

### Hormonal Regulation in the *adgf-a* Mutant

The delayed development and low pupariation rate in the *adgf-a* mutant larvae (see Figures 2A and 7A) could be caused by an effect on hormonal regulation of development. The main source of developmental hormones in the *Drosophila* larva is the ring gland, composed of the prothoracic gland, corpus allatum, and corpus cardiacum [28]. The prothoracic gland releases the steroid molting hormone ecdysone, which is converted to an active form, 20-hydroxyecdysone (20E), by the fat body as well as some of the target organs [29]. The block of pupariation in the *adgf-a* mutant suggested that the level of ecdysone in these larvae might not be sufficient to initiate pupariation. To test this possibility, we tried to rescue the phenotype by feeding mutant larvae 20E, which can initiate pupariation in the *ecd^1^* mutant, which has an extremely low level of ecdysone [30,31]. The results (Figure 7A) clearly demonstrate that the *adgf-a* mutant larvae are responsive to ecdysone and that this treatment restores the pupariation frequency to almost wild-type level. The delay in development is also significantly reduced (Figure 7A).

Since the *adgf-a* mutant shows certain precocious metamorphic changes (macrophage differentiation and fat body disintegration), we speculated that a reduced ecdysteroid level could be caused by precocious degeneration of the prothoracic part of the ring gland. However, the overall structure of the ring gland is not visibly affected even in the oldest larvae (10 d, i.e., 5 d after the heterozygous siblings pupariated) with a fully disintegrated fat body (Figure 7B and 7C).

We also used a transgenic line carrying the *SgsΔ3-GFP* construct, which was previously used to monitor the effects of ecdysteroid levels on glue protein expression in salivary glands [32]. All analyzed *adgf-a* mutant larvae carrying the *SgsΔ3-GFP* construct showed normal expression of Sgs-GFP in salivary glands (Figure 7D). Mutants that pupariated usually showed typical GFP expectoration, indicating the presence of a high premetamorphic peak of ecdysteroids (Figure 7E). In some cases, GFP was secreted into the lumen of salivary glands, but was not expectorated (Figure 7F), which is similar to the defect seen in animals expressing the dominant-negative form of ecdysone receptor driven by the *Sgs3-Gal4* driver [33]. These results demonstrate that the target tissues of *adgf-a* mutants are normally responsive to ecdysteroids and that they are probably capable of releasing ecdysteroids, although the level of ecdysteroids might vary.?>

### ADGF-A Genetically Interacts with Toll Signaling Pathway

The antimicrobial response of *Drosophila* includes at least two distinct signaling pathways [34]—the Toll signaling pathway, which leads to the activation of two nuclear factor kappa B (NF-κB) factors, *Dorsal*-related immunity factor (DIF) and dorsal (DL); and the immune deficiency protein pathway activating the third NF-κB factor, Relish (REL). A zygotic null mutation in *cactus* (*cact;* a *Drosophila* inhibitor of NF-κB) leads to hyperproliferation of hemocytes, melanotic tumor formation, disintegration of fat body, and slower larval development, with 60% larval lethality, as well as a thin body-shape phenotype [35]. All of these phenotypes are strikingly similar to the abnormalities seen in *adgf-a* mutants, which was our first clue as to a possible interaction of ADGF-A with the Toll signaling pathway. We hypothesized that the activity of ADGF-A is suppressed by Toll signaling, resulting in similar phenotypes of the *adgf-a* mutation and constitutive activation of Toll pathway.

To test this hypothesis, we crossed transgenic flies carrying *ADGF-A* gene under the control of a heat-shock promoter on Chromosome II *(HS-ADGF-A)* with *cact^E8^* (a lethal allele of *cact* on Chromosome II, which, in combination with *cact^D13^,* results in a zygotic null combination, or, with *cact^IIIG^,* results in zygotic hypomorphic combination).

Overexpression of ADGF-A in animals with a hypomorphic *cact* combination *(cact^E8^*/*cact^IIIG^)* increased the adult survival rate almost 4-fold (Figure 8A). The rescue could be increased by multiple heat shocks before pupariation to 7-fold (unpublished data). The suppression of melanotic tumor formation is also significant (from more than 80% down to 26%, Figure 8B). The most severe *cact* null mutation *(cact^E8^*/*cact^D13^),* leading to developmental arrest in larvae (less than 8% pupate), is partially rescued in animals with overexpression of ADGF-A when the pupariation rate is increased 3-fold (Figure 8A).

**Figure 8:**
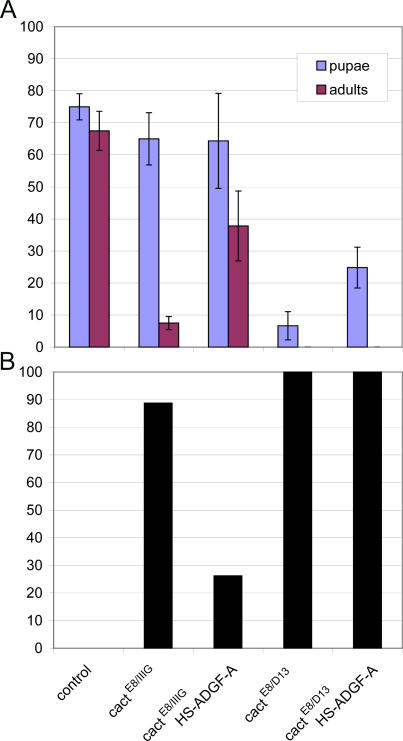
Genetic Interactions of Toll Signaling and ADGF-A Survival rate and melanotic tumor formation were compared in mutants in the Toll signaling pathway and in similar mutants with overexpression of ADGF-A using the *HS-ADGF-A* construct. (A) The bar graph shows the percentage of the pupae (blue bars) and adult flies (purple bars) demonstrating the larval and pupal survival of each genotype. The *x*-axis shows the genotypes and is shared with (B). Flies heterozygous for the *cact* mutation were used as a control. (B) Percentage of late third instar larvae presenting melanotic tumor formation.

These results demonstrate that ADGF-A overexpression can partially rescue the effects of constitutively active Toll signaling in larvae, mainly the developmental arrest, but also the melanotic tumor formation, in the case of hypomorphic *cact* mutants.

## Discussion

### ADA Deficiency in *Drosophila* Causes Abnormal Hemocyte Development, Melanotic Tumor Formation, Fat Body Degeneration, and Delayed Development

We have established an ADA deficiency model in *Drosophila* in order to study the effects of altered adenosine levels in vivo. We produced a loss-of-function mutation in the *ADGF-A* gene, which produces a product (ADGF-A) with ADA activity. When homozygous, the mutation causes abnormal hemocyte development, leading to melanotic tumor formation [36], as well as fat-body disintegration associated with death during the larval stage or delayed transition to the pupal stage of development. In agreement with our previous study using cells cultured in vitro [16], here we have shown that ADA enzymatic activity is essential for ADGF-A function in vivo, when this function is assayed by testing for rescue of the mutant phenotype. Just as increased levels of both ADA substrates, adenosine and deoxyadenosine, are found in blood of SCID patients [5], *adgf-a* mutant larvae also have elevated levels of adenosine and deoxyadenosine, indicating that the mutant phenotype is caused by disturbance in the turnover of these nucleosides.

Expression of ADGF-A only in the lymph glands is sufficient to fully rescue the mutant phenotype, indicating that the hemocytes within the lymph glands play a major role in regulation of adenosine levels in the hemolymph. A similar regulatory role has also been attributed to blood cells in humans [5]. This suggests a function for ADGF-A within the lymph gland. However, ADGF-A behaves as a soluble growth factor and could be released from the lymph gland to activate targets elsewhere in the larval body. Our results show that ADGF-A functions by limiting the level of extracellular adenosine, and in this way the protein could have a systemic function even if it were restricted to its tissue of origin. Although our tests did not exclude a role for ADGF-A in circulating hemocytes (which constitute a separate lineage from the lymph gland hemocytes [20]), we showed that expression of ADGF-A in circulating hemocytes is not required for rescue of the *adgf-a* mutant phenotype, since *e33C-Gal4/UAS-ADGF-A*—which expresses ADGF-A in the lymph gland but not in circulating hemocytes—fully rescued the phenotype.

### ADGF-A Is Involved in Hemocyte Differentiation in the Lymph Glands

Late third-instar larvae homozygous for the *adgf-a* mutation contain, on average, seven times more hemocytes in circulation than wild-type larvae, and most of these cells show strong adhesive properties compared to normal larval plasmatocytes, which remain rounded after settling down on the substrate. Although these cells share other characteristics with plasmatocytes, they are normally not seen in circulation until they are released from the lymph glands at the onset of metamorphosis under the regulation of ecdysone to serve as phagocytes for histolysing tissues during metamorphosis—thus, they are referred to as pupal macrophages [19]. In agreement with the presence of these cells in circulation, at least the first lobes of the lymph glands are usually completely dispersed in late third-instar mutant larvae. This indication of precocious metamorphic changes [36] in the mutant is further supported by the finding that hemocytes aggregate in a segmental pattern in early rather than late third instar (see Figure 4H–4J), and that the hemocytes lose expression of Hemolectin in late third-instar larvae rather than at the onset of metamorphosis (see Figure 4G) [21].

Recent studies show that the Toll signaling pathway, which is already known to be involved in the control of innate immunity of both *Drosophila* and mammals [34], may also be involved in the control of hemocyte differentiation in the *Drosophila* larva. Constitutive activation of Toll signaling leads to developmental arrest and hematopoietic defects associated with melanotic tumor formation [35], similar to the phenotype of the *adgf-a* mutant. Our work also shows that forced expression of the *ADGF-A* gene can rescue the effects of overactive Toll signaling, suggesting that ADGF-A might function downstream of Toll signaling to control its effects. This conclusion is consistent with the existence of a putative binding site for Dorsal (one of two known effectors of Toll signaling) in the *ADGF-A* promoter (Figure 9). It will be important to explore this connection further, since recent studies suggest an interaction between adenosine signaling and the NF-κB signaling pathway, which is the mammalian counterpart of the Toll pathway [37].

**Figure 9:**
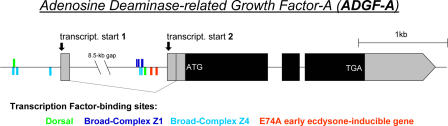
Schematic Map of the *ADGF-A* Gene with Promoter Analysis The *ADGF-A* gene contains four exons and two transcriptional starts [17,47]. We analyzed sequences preceding both transcriptional starts for the presence of known transcriptional factor binding sites using the software program Gene2Promoter (Genomatix Software GmbH). Selected sites are represented by color bars in approximate positions of promoter regions. The legend under the sequence show the names of transcription factors binding to matching colored binding sites.

### Precocious Fat-Body Disintegration Caused by Mutant Hemocytes

One of the most remarkable features of the *adgf-a* mutant phenotype is the disintegration of the fat body in third-instar larvae, another indication of precocious metamorphic changes since the disintegration normally occurs much later, during pupal life. Furthermore, our study of this mutant provides strong evidence that the fat body disintegration is promoted by the action of hemocytes. Fat body disintegration was significantly suppressed when the hemocyte number was reduced using the *l(3)hem^1^* mutation [26], and fully blocked by the *croquemort* (*crq*) mutation [27] which affects a CD36-related receptor (Croquemort) expressed on macrophages and required in phagocytosis of apoptotic cells. Human CD36 is a scavenger receptor which, in combination with the macrophage vitronectin receptor and thrombospondin, binds apoptotic cells. A similar role of Croquemort for removing histolysing tissues during *Drosophila* metamorphosis has not yet been tested, but seems likely since the *crq* mutant used in this study *(crq^KG01679^)* is lethal in pupae.

The idea that hemocytes are involved in fat body dissociation in *Drosophila* is further supported by work on the flesh fly *Sarcophaga*. Natori's group showed that proteinase cathepsin B was released from pupal hemocytes when they interacted with the fat body, and that this enzyme digested the basement membrane of the fat body, causing the tissue to dissociate [38,39]. They also showed that the interaction of hemocytes with the fat body is mediated by a 120-kDa membrane protein localized specifically on pupal hemocytes [40]. This protein was suggested to be a scavenger receptor, but it does not seem to be homologous to *Drosophila* Croquemort (unpublished data). Work by Franc et al. [27] is consistent with the idea that more than one scavenger receptor is involved in this process.

### Possible Signaling Role for Adenosine

The precocious metamorphic changes that appear to occur in response to elevated adenosine in the *adgf-a* mutant larvae lead to the suggestion that adenosine may act as a regulatory signal for these processes during normal development. One possibility is that adenosine acts as a downstream effector of ecdysone-regulated prepupal changes, and that the increase in adenosine concentration is mediated by ecdysone-induced down-regulation of ADGF-A expression. This is supported by the presence of multiple sites for ecdysone-inducible transcription regulators in the *ADGF-A* promoter (Figure 9). Adenosine could serve as a signal for macrophage differentiation, and the lack of adenosine deaminase activity due to the *adgf-a* mutation could cause precocious differentiation of these cells in mutant larvae. We are now carrying out direct tests of the idea that the differentiation of hemocytes in mutant larvae is caused by elevated adenosine. If confirmed, this effect would have general significance, since in ADA-deficient mice, inflammatory changes in the lungs include an accumulation of activated alveolar macrophages [41], and this could also be mediated by elevated adenosine.

### Elevated Adenosine Delays Development and Inhibits Pupariation

The elevated adenosine in the *adgf-a* mutant larvae leads to precocious changes (hemocyte differentiation and fat body disintegration) resembling those normally occurring at the time of metamorphosis, but it also is associated with an apparently opposite effect, in that it causes a significant delay in progress through the third larval instar and a decrease in the frequency of successful pupariation (formation of the puparium from the larval cuticle), which is one of the earliest steps in metamorphosis. We conclude that the mutation has additional effects on the hormonal regulation of development.

One possible explanation for the developmental delay and failure to pupariate is that the *adgf-a* mutation affects the production or release of ecdysteroid hormones from the major endocrine organ of the *Drosophila* larva—the ring gland. This is supported by the fact that pupariation rate and survival of the *adgf-a* mutant can be significantly improved by expression of transgenic ADGF-A in the ring gland and salivary glands. We suggest that this somehow interferes with the regulation of hormone release. Other mutants with hormonal dysregulation show delayed larval development and failure to pupariate [42,43]. Presumably the elevated adenosine in the *adgf-a* mutant blocks the production or release of ecdysone from the ring gland by an unknown mechanism. This idea is supported by our finding that both pupariation rate and survival of the *adgf-a* mutant can also be improved by feeding the mutant larvae with 20E in the diet (see Figure 7A). Thus it is clear that the *adgf-a* mutant is arrested in development due to an effect of the mutation on hormone production from the ring gland.

The arrest of development in the *adgf-a* mutants was significantly suppressed by loss of the adenosine receptor caused by the *adoR* mutation: larvae simply homozygous for *adgf-a* pupated after two or more days, whereas larvae also homozygous for *adoR* pupated within 1 d after their heterozygous siblings (see Figure 7A). Therefore, adenosine signaling through the AdoR must play a role in the developmental arrest of the *adgf-a* mutant, and this is most likely mediated by signaling to the ring gland, where AdoR is expressed (ED, unpublished data). The mutation in AdoR does not block macrophage differentiation and fat-body disintegration, so this effect must involve another, as yet uncharacterized mechanism independent of AdoR signaling. Work using adenosine-receptor deficient mammalian cells also suggested the existence of a novel, undefined adenosine signaling mechanism [44]. However, we cannot exclude the role of elevated deoxyadenosine in these effects. *Drosophila,* now with the advantage of the well-characterized *adgf-a* mutant, could serve as an ideal model system in which to investigate this mechanism.

### Concluding Remarks

In our previous work using cells cultured in vitro, we showed that, as in mammals, adenosine can block proliferation and/or survival of some *Drosophila* cell types [16]. In the present work, we have established a *Drosophila* model to study altered levels of adenosine and deoxyadenosine in vivo, and we have shown that loss of *ADGF-A* function causes an increase of these nucleosides in larval hemolymph. Although the *adgf-a* mutation leads to larval or pupal death, we have shown that this is not due to the adenosine or deoxyadenosine simply blocking cellular proliferation or survival, as the experiments in vitro would suggest. Rather, this mutation leads to an increase in number of hemocytes at the end of larval development due to the differentiation and release of hemocytes from the lymph glands. Hemocytes also differentiate and are released from the lymph glands during systemic infection [19]. Together with our result suggesting an interaction between Toll signaling and ADGF-A, this leads to the hypothesis that adenosine controls hemocyte differentiation in response to infection, and that it signals through the adenosine receptor to postpone the next developmental step, metamorphosis. This would be consistent with the role of adenosine as a “stress hormone” in mammals [6]. A similar process of hemocyte differentiation and release from the lymph glands normally takes place at the onset of metamorphosis, when pupal macrophages remove histolyzing tissues. The *ADGF-A* promoter contains consensus binding sites for effectors of both Toll and ecdysone signaling. This raises the possibility that adenosine plays a role in the control of metamorphosis as well as in the response to stress.

## Materials and Methods

### Fly strains and genetics

For standard procedures, flies were raised at 25 °C on a standard cornmeal-agar-yeast-molasses diet supplemented with 0.3% Nipagin to retard mold growth. Oregon flies were used as the wild-type *Drosophila* strain, but in most cases the *y w* strain was used as a control since most mutations were carried in the *y w* background. A mutation in the *ADGF-A* gene on Chromosome III was obtained as described earlier [17]. In this study, the mutation described as *adgf-a^karel^* was used in all experiments and is referred to here as *adgf-a*. A mutation in the adenosine receptor gene on Chromosome III was produced by the ends-out targeting method (ED, unpublished data) and is referred to here as *adoR*. Transgenic flies carrying *HS-ADGF-A,* *UAS-ADGF-Amyc,* and *UAS-mutADGF-Amyc* construct (see description below) were produced by a modified P-element transformation method [45]. *HS-ADGF-A,* *UAS-ADGF-Amyc[2A],* *UAS-ADGF-Amyc[7A],* *UAS-mutADGF-Amyc[1A],* and*UAS-mutADGF-Amyc[3B],* all insertions on Chromosome II, were isolated and used in this work. The following markers and mutations were obtained from the Bloomington stock center, accessible at http://fly.bio.indiana.edu/ (stock numbers provided in parentheses): *Hml-GFP* marker (Hml-Gal4/UAS-GFP) expressing GFP in embryonic and larval hemocytes on Chromosome II (BL-6397), the *l(3)hem^1^* mutation on Chromosome III (BL-6184), and the *crq^KG01679^* mutation in the *crq* gene on Chromosome II (BL-14900). Mutants in Toll signaling pathway were obtained from Dr. S. Govind: *cact^E8^,* *cact^IIIG^,* and *cact^D13^* mutations in the *cact* gene on Chromosome II. The Gal4/UAS [18] system was used for protein misexpression. The following were obtained from the Bloomington stock center (stock numbers in parenthesis): *Cg-Gal4* on Chromosome II (BL-7011), *Pw^+mW.hs^=GawB5015* on II (BL-2721), *Pw^+mW.hs^=GawBc564* on II (BL-6982), *Pw^+mW.hs^=GawBT110* on II (BL-6998), *Hml-Gal4* on II (BL-6396), *Dot-Gal4^43A^* on X (BL-6903), *Dot-Gal4^11C^* on II (BL-6902), and *Lsp2-Gal4* (BL-6357) on III. The *Pen2.4-GAL4e33C* lethal insertion on Chromosome III was obtained from Dr. N. Perrimon's lab, and the *Dmef2-Gal4* driver on II from Dr. A. Michelson. Expression information of these Gal4 drivers is provided in Table 1. A stock carrying the ubiquitous actin-Gal4 driver (*P-actin-Gal4 UAS-GFP/CyO;* lethal insertion on Chromosome II) was obtained from Dr. R. Sousa. To recognize homozygous larvae, balancer chromosomes with the GFP marker were used: *CyO Pw^+mW.hs^=Ubi-GFP.S65TPAD1* (BL-4559) and *TM3 Pw^+mC^=ActGFPJMR2 Ser* (BL-4888). Transgenic flies SgsGFP-1 (insertion on Chromosome X) and SgsGFP-2 (insertion on Chromosome II) containing the chimeric gene construct SgsΔ3-GFP were obtained from Dr. A. J. Andres. For expression of ADGF-A using the *HS-ADGF-A* construct, flies were heat shocked as late embryos/early first instars at 37 °C for 30 min. In all rescue experiments, 30 freshly hatched homozygous first-instar larvae were selected using a GFP dissecting microscope and transferred into fresh vials (at least four vials for each variant). They were left to develop at 25 °C and examined as wandering third-instar larvae, pupae, and adults.

### Ecdysone treatment

Mutant larvae were raised on plates with yeast paste at 25 °C and transferred to vials with glucose-yeast medium (control) or with glucose-yeast medium containing 20-hydroxyecdysone (H-5142; Sigma-Aldrich, St. Louis, Missouri, United States) at a concentration of 0.5 mg/ml shortly after the L2/L3 molt. Numbers of puparia were counted at 12-h intervals after the 120-h time point (when the first control larvae start to pupariate). The *ecd^1^* flies (Bloomington stock BL-218) served as a control for the functional 20E diet [31]: flies were raised at 22 °C (permissive temperature for the temperature-sensitive *ecd^1^* mutation) and transferred to vials with control or 20E-containing diet and raised at 29 °C (restrictive temperature).

### Fat body observation

Living late third-instar larvae were washed and examined in PBS using a standard dissecting microscope with transmitted light. For finer analysis, the fat body was dissected from larvae in PBS and observed using a dissecting microscope. GFP-stained fat body was observed in living, etherized larvae in PBS solution on a standard microscopic slide with a coverslip under a fluorescence microscope.

### Hemocyte counts and observations

Circulating hemocytes were obtained by opening two late third-instar larvae in 30 μl of PBS. This allowed us to collect all hemolymph from the larvae in a defined volume. The solution with circulating hemocytes was mixed by gently pipetting, and part was transferred into the chamber of an improved Neubauer hemocytometer. Cell number was recounted to one animal equivalent. Hemocyte morphology was observed by differential interference contrast microscopy of living cells in Shields and Sang Insect Medium (Sigma-Aldrich) obtained by the same procedure as for counting. To observe hemocyte morphology, samples were analyzed at least 10 min after the deposition of solution with hemocytes, in order to allow the cells to adhere to the surface of the slide. Crystal cells were visualized by heating larvae at 60 °C for 10 min in a water bath [46]. GFP-stained hemocytes were observed in living, etherized larvae in PBS solution on a standard microscopic slide with a coverslip under the fluorescence microscope or by deposition of hemocytes in PBS as for counting and observing under the fluorescence microscope.

### Transgenic constructs

Wild-type cDNA for *ADGF-A* was amplified by PCR using proofreading DNA polymerase (ProofStart; Invitrogen, Carlsbad, California, United States) from a pOT2 vector containing the *ADGF-A* EST-clone (GH08276) using the following primers: 5′- CGTCTAGAATGTCGCCAGTCATCCGCC-3′ (5′ end primer with XbaI tail) and 5′- GCTGATCATCAATCGATCCGTTGACTGGGGGA-3′ (3′ end primer with BclI tail). The PCR product was cloned into the pGEM-T Easy vector (Promega, Madison, Wisconsin, United States), and the resulting plasmid *(ADGF-A-pGEM)* was cut by NotI/SpeI restriction enzymes. The ADGF-A fragment was then cloned into the pCaSpeR-hs) vector cut by NotI/XbaI to obtain the *HS-ADGF-A* construct. The myc tag was added to the C terminus of the ADGF-A protein for detection by anti-c-Myc antibody (Sigma-Aldrich). To produce a *UAS-ADGF-Amyc* construct, the ADGF-A fragment was amplified (by ProofStart from pOT2 vector) using the following primers: 5′- AATCTCGAGCTCATCATGTCGCCAGTCATC-3′ (5′ end with XhoI tail) and 5′- TATCTAGATCGATCCGTTGACTGGGGG-3′ (3′ end with XbaI tail). The fragment was cut by XhoI/XbaI and cloned into the pUAST vector modified by MZ. The sequence encoding the myc-tag 5′- GAGCAAAAGCTCATTTCTGAAGAGGACTTG-3′ plus a stop codon was inserted into XbaI site of pUAST (using the XbaI site on the 5′ end and the NheI site on the 3′ end) cut by XhoI/XbaI. A mutated version—*UAS-mutADGF-Amyc*—was prepared in the same way as *UAS-ADGF-Amyc,* but pBLUESCRIPT containing mutated *ADGF-A* cDNA was used as a template. The mutated version of ADGF-A (carrying a mutation causing the substitution of two amino acids—His386 and Ala387 for Glu and Leu, respectively) in the catalytic domain, shown to abolish adenosine deaminase activity [16], was prepared by recombinant PCR using the following recombinant primers: 5′- TCTACTTCGAGCTCGGAGAAACAAACTGGTTCGGT-3′ and 5′- CTCCGAGCTCGAAGTAGAAATCAATGTCATCG-3′ and the same 5′ and 3′ end primers as above.

### Adenosine and deoxyadenosine concentrations measurement

The detection method used liquid chromatography and mass spectrometry (LC/MS method) of deproteinated hemolymph samples. Larval hemolymph was collected from several larvae and centrifuged to pellet the hemocytes. 1 μl of hemolymph was diluted in 99 μl of buffer. The sample was introduced in CH_3_CN-0.05% TFA (50:50) either via a syringe pump at 3 μl/min or via an RP-C18 150 mm × 1 mm Symmetry C_8_ column at 50 μl/min employing an LCQ electrospray ion source operated at 4.2 kV. The peaks were then identified using the electrospray MS^N^ mass spectra obtained by the collision-induced decomposition of the MH^+^ ion and its product ions in a series of MS^N^ experiments that were performed with the ion trap mass spectrometer. The sugar moiety was cleaved off the adenosine molecule and produced ion with a molecular weight of 136 (adenine), which was then detected by MS.

## Supporting Information

### Accession Numbers

The FlyBase (http://flybase.bio.indiana.edu/) accession numbers for the genes and proteins discussed in this paper are: *Ada* (FBgn0037661), ADGF-A (FBgn0036752), *ADGF-A* cDNA (FBtp0018801), *adgf-a* mutation (FBal0157461), *adgf-a^karel^* (FBab0038650), ADGF-D (FBgn0038172), AdoR (the *CG9753* gene; FBgn0039747), *c564-Gal4* (FBti0002592), *cact^D13^* (FBal0001509), *cact^E8^* (FBal0030706), *cact^IIIG^* (FBal0001515), *cactus* (FBgn0000250), *Cg-Gal4* (FBtp0012452) , *croquemort* (FBgn0015924), *crq^KG01679^* (FBal0147219), *Dmef2-Gal4* (FBtp0006434), Dorsal (FBgn0000462), *Dorsal*-related immunity factor (FBgn0011274), *Dot-Gal4^11C^* (FBti0024024), *Dot-Gal4^43A^* (FBti0024023), *e33C-Gal4* (FBti0002599), *ecd^1^* (FBal0003500), *GawB^5015^* (FBti0001256 ), Hemolectin (FBgn0029167), *HS-ADGF-A* (FBtp0018800), *l(3)hem^1^* (FBal0010873), *Lsp2-Gal4* (FBti0018531), Relish (FBgn0014018), *SgsΔ3-GFP* (FBtp0013370), *Sgs3-Gal4* (FBtp0016397), and *T110-Gal4* (FBti0002605).

The GenBank Nucleotide database accession numbers for the genes and proteins discussed in this papers are: cathepsin B (496316), *IDGF* (1402633), and pupal hemocyte 120-kDa membrane protein (7023974).

HUGO Gene Nomenclature Committee (HGNC; http://www.gene.ucl.ac.uk/nomenclature/) accession numbers for the genes and proteins discussed in this paper are: *CECR1* (1839) and human CD36 (1663).

Online Mendelian Inheritance in Man (OMIM; http://www.ncbi.nlm.nih.gov/entrez/query.fcgi?db=OMIM) accession numbers for the conditions discussed in this paper are: cat eye syndrome (115470) and SCID (102700).

## Abbreviations

20E: 20-hydroxyecdysone
ADA: adenosine deaminase
ADGF-A: adenosine deaminase-related growth factor-A
AdoR: Drosophila adenosine receptor
cact: cactus
crq: croquemort
GFP: green fluorescent protein
HML: Hemolectin
l(3)hem: l(3)hematopoiesis missing gene
NF-κB: nuclear factor kappa B
SCID: severe combined immunodeficiency disease
